# Spatiotemporal 22q11.21 Protein Network Implicates DGCR8-Dependent MicroRNA Biogenesis as a Risk for Late Fetal Cortical Development in Psychiatric Diseases

**DOI:** 10.3390/life11060514

**Published:** 2021-05-31

**Authors:** Liang Chen, Wenxiang Cai, Weidi Wang, Zhe Liu, Guan-Ning Lin

**Affiliations:** School of Biomedical Engineering, Shanghai Jiao Tong University, Shanghai 200030, China; chenliang66@sjtu.edu.cn (L.C.); caiwenxiang@sjtu.edu.cn (W.C.); wwd-swxx@foxmail.com (W.W.); liuzlm1030@sjtu.edu.cn (Z.L.)

**Keywords:** CNV, PPI, spatiotemporal network, chromosome 22q11.21, DGCR8

## Abstract

The chromosome 22q11.21 copy number variant (CNV) is a vital risk factor that can be a genetic predisposition to neurodevelopmental disorders (NDD). As the 22q11.21 CNV affects multiple genes, causal disease genes and mechanisms affected are still poorly understood. Thus, we aimed to identify the most impactful 22q11.21 CNV genes and the potential impacted human brain regions, developmental stages and signaling pathways. We constructed the spatiotemporal dynamic networks of 22q11.21 CNV genes using the brain developmental transcriptome and physical protein–protein interactions. The affected brain regions, developmental stages, driver genes and pathways were subsequently investigated via integrated bioinformatics analysis. As a result, we first identified that 22q11.21 CNV genes affect the cortical area mainly during late fetal periods. Interestingly, we observed that connections between a driver gene, *DGCR8*, and its interacting partners, *MECP2* and *CUL3*, also network hubs, only existed in the network of the late fetal period within the cortical region, suggesting their functional specificity during brain development. We also confirmed the physical interaction result between DGCR8 and CUL3 by liquid chromatography-tandem mass spectrometry. In conclusion, our results could suggest that the disruption of DGCR8-dependent microRNA biogenesis plays a vital role in NDD for late fetal cortical development.

## 1. Introduction

Copy number variants (CNVs) are duplications or deletions of a genomic fragment ranging from one kilobase (Kb) to five megabases (Mb) [1]. They have often been identified as risk factors for genetic disorders [2]. The chromosome 22q11.2 region includes low copy repeats (LCRs) that mediate nonallelic homologous recombination. More specifically, the most commonly 22q11.2 deleted or duplicated region spans LCR-A to LCR-D, located on chromosome 22q11.21 [3]. Previous studies showed 22q11.21 deletion to be associated with several psychiatric disorders. 22q11.2 deletion syndrome is also known as DiGeorge or velocardiofacial syndrome. An elevated rate of autism spectrum disorder (ASD) has been reported in patients with 22q11.2 deletion syndrome [3]. In addition, deletion of 22q11.21 can cause schizophrenia, intellectual delay or attention deficit hyperactivity disorder [4,5], and duplication of 22q11.21 may lead to learning disability, developmental delay and ASD [3,6].

Previous genetic studies suggested that several 22q11.21 genes might be involved in psychiatric disorders [7,8]. In an attempt to pinpoint the role of 22q11.21 CNV genes in neurodevelopmental disorders, animal models have been established, and the biological functions of these genes have been examined [9]. One clinical phenotype, microcephaly, has often been observed in mental disorder patients with 22q11.21 deletion [10,11]. This phenotype has been reproduced in DGCR8 knockout zebrafish and mouse models [12,13]. Although these phenotypes have been observed, the underlying genetic mechanisms are still unclear. As a component of the microprocessor complex, DGCR8 is responsible for processing long primary miRNAs (pri-miRNAs) into short hairpins called precursor miRNAs (pre-miRNAs). It was reported that microRNAs (miRNAs) play a pivotal role in ASD and schizophrenia. In addition, another 22q11.21CNV gene, Ran-binding protein 1 (RANBP1), plays a critical role in RAN-dependent nucleocytoplasmic transport [14]. Homozygous RANBP1 mutant embryos exhibited microcephaly [15]. Previous studies suggested that RANBP1 is involved in nucleocytoplasmic transport to regulate neuronal polarity [16]. Although the biological functions of the individual gene have been uncovered, little is known about how the 22q11.21 CNV causes neurodevelopmental disorders since these multiple genes play different roles across different anatomic structures during different developmental stages.

Protein–protein interactions (PPIs) play an important role in biological processes. PPI network analysis is especially useful for discovering the underlying molecular mechanism in systems biology [17,18]. Actually, a protein interaction network frequently describes physical PPIs between proteins [19]. Analyses of molecular networks can identify the biological module and complex signaling pathways [20]. Many studies explored the pathogenesis of CNVs in psychiatric disorders by constructing a static topological network [21,22]. However, protein expression is dynamic, which can differ within various anatomical structures and developmental stages, and within the protein interactions as well [23,24,25]. 

Previous studies showed strong correlations between higher co-expression and protein interaction [26]. PPIs change along with dynamic expression levels of proteins. For this reason, PPIs could be affirmed by co-expression data. Therefore, integrating PPIs with gene expression data can uncover protein interactions at different developmental periods and in different anatomical areas. Previous works revealed the pathogenesis of candidate genes or CNVs by constructing spatiotemporal PPI networks due to alterations of protein expression patterns in different anatomical areas and during different developmental stages [27,28]. Although significant progress has been made [29], the particular human brain regions, periods, protein networks and signal pathways influenced by the 22q11.21 CNV remain unclear. Thus, in this study, we constructed a spatiotemporal network of the 22q11.21 CNV by integrating data from the human brain developmental transcriptome with physical interactions of 22q11.21 proteins. Our results demonstrate that 22q11.21 proteins interact with their related partners significantly in three particular spatiotemporal intervals, and the interaction patterns alter across these intervals. In particular, we identified that the parietal, temporal and occipital lobes are critical regions for the interactions between 22q11.21 proteins and their partners during early mid-fetal and late fetal periods. Furthermore, we observe that DGCR8 interacts with MECP2 and CUL3 during the late fetal period. Our results suggest that the DGCR8-dependent microRNA biogenesis pathway is crucial for the 22q11.21 CNV genes involved in psychiatric disorders.

## 2. Materials and Methods

### 2.1. Identification of 22q11.21 Genes and the Human Brain Transcriptome Data Collection

We collected data from previous studies that assessed the 22q11.21 CNV located on chromosome 22 (chr: 22, 17.9–20.5) [3,30]. Twenty-six genes are located on this region (Supplementary Table S1). Human brain transcriptome data were downloaded from BrainSpan (http://www.brainspan.org, RNA-Seq Gencode v3c summarized to genes). BrainSpan provides normalized reads per kilobase per million (RPKM) expression data on 578 developing brain samples across 13 developmental stages. The expression values for samples of the same age and from the same area were averaged. To reduce noise, we removed genes with a log2 intensity of <0.4 in all samples and with a coefficient of variation of <0.07. Therefore, 15,095 genes were retained for analysis.

### 2.2. The Datasets of Physical Protein–Protein Interactions Restricted to Brain-Expressed Genes

Protein–protein interaction data were downloaded from the BioGRID database (https://downloads.thebiogrid.org/BioGRID/Release-Archive/BIOGRID-3.4.161/) (accessed on 19 May 2018). BIOGRID-ORGANISM-3.4.161.tab2 was downloaded in May 2018. The human PPIs were utilized (BIOGRID-ORGANISM-Homo_sapiens-3.4.161.tab2.txt). We obtained only physical protein–protein interactions. Redundancy and self-interaction data were also removed, leaving 241,123 pairs. Next, the protein–protein interaction (PPI) network was integrated with the human brain transcriptome to assemble a brain-expressed human interactome, termed HIBE.

### 2.3. Construction of Spatiotemporal Protein Network

We defined eight non-overlapping periods (Supplementary Table S2). Anatomical structures were divided into four areas according to anatomical and functional similarity (Supplementary Table S3). Consequently, we constructed thirty-one spatiotemporal protein networks after removing one region from P3 (P3R4) due to a lack of enough RNA-seq data. CNV genes were mapped to the HIBE network to build a static network. Spatiotemporal expression data were combined with static PPI networks, and the SCC (Spearman correlation coefficient) values were calculated. The interactions were confirmed only if the SCC was >0.5. Thirty-one networks were constructed.

We defined eight developmental periods as previously described (Supplementary Table S2) [28,31]. Anatomical structures were divided into four areas as previously described [28] (Supplementary Table S3) according to anatomical and functional similarity. Consequently, we constructed thirty-one spatiotemporal protein networks after removing one region from P3 (P3R4) due to a lack of enough RNA-seq data. CNV genes were mapped to the HIBE network to build a static network. Spatiotemporal expression data were combined with static PPI networks, and the SCC (Spearman correlation coefficient) was calculated. The interactions were confirmed only if the SCC was >0.5. We used Cytoscape software for network visualization. Thirty-one networks were constructed.

### 2.4. Enrichment Analyses in Three Spatiotemporal Networks

Fractions of co-expression interacting pairs were calculated from 22q11.21 proteins and three control datasets. The Fisher exact test was used to identify significant enrichment of connectivity for the 22q11.21 CNV. One-way ANOVA tests were performed to analyze the difference between 22q11.21 networks from the same developmental period (P2R1, P2R2) or the same anatomical area (P2R1 and P4R1). Topological features were defined for each 22q11.21 CNV gene: the fraction of interacting partners unique to one network and the fraction of interacting partners shared by two networks (Supplementary Tables S4 and S5). The statistically significant differences were calculated using ANOVA tests, and genes from dynamic networks were analyzed using Metascape [32]. Functional enrichment was performed in three GO categories: biological process, molecular function and cellular component. Terms with *p* < 0.01, a minimum count of 3 and an enrichment factor of >1.5 (the enrichment factor was defined as the observed count’s ratio to the count expected by chance) were collected and grouped into clusters based on their membership similarities. Furthermore, *p*-values were calculated based on the cumulative hypergeometric distribution. The Q-value was calculated using Benjamini–Hochberg correction for multiple testing.

ASD risk genes’ associated genes were from a previous report. The FMRP target gene set was from a previous publication [33]. Voltage-gated calcium channel complex proteins were from a previous study by Catrin Swantje Müller [34,35]. Developmental delay genes were derived from a previous report [36]. Two gene sets were downloaded from the Mouse Genome Informatics (MGI) database (http://www.informatics.jax.org), abnormal nervous system electrophysiology (MP: 0002272) and abnormal long-term potentiation (MP: 0002207) [34]. Differences between the mutations of proteins from 22q11.21 spatiotemporal networks and mutations from 20,240 genes were analyzed using Fisher’s exact test. The *p*-values were corrected using the Benjamini–Hochberg method. To test whether proteins from 22q11.21 spatiotemporal networks are enriched in two gene sets (MP: 0002272, 0002207), Fisher’s exact test was utilized. The Benjamini–Hochberg method was used to correct the *p*-values.

### 2.5. Cell Culture and Transfection

HEK293T cells were cultured in Dulbecco’s Modified Eagle Medium (DMEM) supplemented with 10% Fetal Bovine Serum and 1% penicillin-streptomycin and maintained in a humidified incubator at 37 °C in an atmosphere containing 5% CO_2_. For cell transfection, 1.5 × 10^6^ cells were seeded into a 10cm dish until they reached 80–90% confluency. Transfections were undertaken using the jetPRIME Transfection Reagent with pCMV6-entry-HA-DGCR8. HEK293T cells were transfected with pCMV6-entry-HA-DGCR8. A total of 10 μg of DNA and 20 μL of transfection reagent were used per 10cm dish. After 48 h, the cells were rinsed with ice-cold PBS, collected and resuspended in lysis buffer (20 mM Tris-Cl, 5 mM EDTA pH 7.4, 150 mM NaCl, 1% Triton X-100 and 10% (vol/vol) glycerol), supplemented with 1mM PMSF, and complete protease inhibitor cocktail. Of the supernatant, 5% was saved for the input control, and the rest of the cell lysates were immunoprecipitated with either anti-HA or control mouse IgG for 12 h at 4 °C. After that, the cell lysates were added to the protein G beads overnight at 4 °C, and immunocomplexes were washed three times with lysis buffer, boiled in 5 × SDS loading buffer with 20mM DTT and then resolved by 10% SDS-PAGE gels (Supplementary Figure S2). The gels were stained with Coomassie brilliant blue (CBB). Protein bands were excised at around 88 kDa.

### 2.6. Peptide Preparation and LC-MS/MS

First, gels were de-stained with 50% (*v*/*v*) methanol and vortexed vigorously for 30 min. Then, gel pieces were washed in water for 15 min. Gel pieces were washed in water for 15 min. Gel pieces were then dehydrated in 100% acetonitrile for 10 min and dried in a vacuum centrifuge. The disulfide bonds of proteins were then reduced with dithiothreitol (10 mM) and alkylated with iodoacetamide (55 mM). Next, gel pieces were washed with 50% (*v*/*v*) acetonitrile and NH_4_HCO_3_ (25 mM) and dehydrated with 100% acetonitrile. Gel pieces were digested with trypsin in NH_4_HCO_3_ (25 mM). Peptides were extracted with 50% (*v*/*v*) acetonitrile and 1% (*v*/*v*) trifluoroacetic acid. Free peptides were dried using a vacuum centrifuge, separated using liquid chromatography (LC) (Easy-nLC 1000; Thermo Fisher, Waltham, MA, USA) and introduced into a Q Exactive mass spectrometer (Thermo Fisher). Finally, peptides were analyzed by MASCOT (www.matrixscience.com).

### 2.7. Proteome Analyses

Data analyses were undertaken using Proteome Discoverer 1.4 (Thermo Scientific), which incorporates the MASCOT search engine. The Homo sapiens database from Uniprot was downloaded on 15 May 2019, and human protein sequences were searched. Carbamidomethyl was used as the fixed modification, with oxidation as the dynamical modification. The maximum number of missed cleavages considered was two. Immunoprecipitation samples were prepared in three independent experiments. Analyses involved only proteins that were detected by MS at least twice.

## 3. Results

### 3.1. Construction of Spatiotemporal Interaction Network for 22q11.21

**Figure 1:**
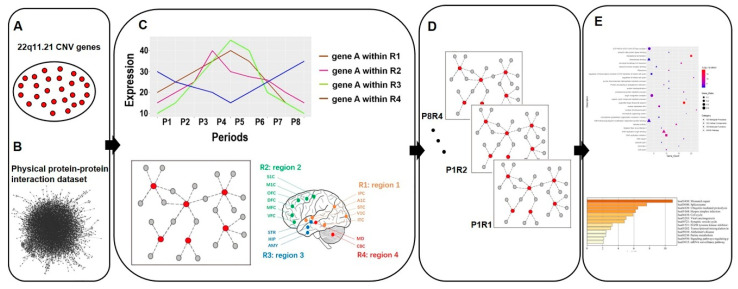
The flow chart shows the pipeline of this research study. (**A**) Twenty-six 22q11.21 CNV genes expressed in the brain were identified. (**B**) Physical protein–protein interaction (PPI) dataset was combined with 22q11.21 CNV genes to construct CNV protein–protein interactions (PPIs). (**C**) 22q11.21 CNV PPIs were combined with the human brain transcriptome dataset [28]. (**D**) A 22q11.21 spatiotemporal co-expression PPI network was established. (**E**) Gene Ontology and pathway analysis were performed.

PPIs occur only if proteins express at the same cell component simultaneously [37]. Multiple studies have reported a robust correlation between co-expression and protein interaction [26,38]. Hence, the combination of data from gene expression and protein interaction could uncover protein interactions at different developmental stages and within various anatomical regions. To study the regulatory role of the 22q11.21 CNV during brain development, we extracted 26 genes located in the chromosomal region of 22q11.21 encompassing ~4.3 Mb (chromosome 22: 17.4–21.7 Mb) (Supplementary Table S1) and constructed dynamic networks by integrating spatiotemporal RNA expression data with 22q11.21 physical PPIs (Figure 1).

Human developmental brain gene expression data were obtained from BrainSpan (www.brainspan.org). Next, we partitioned the expression data by their developmental periods and brain regions as previously described [28] (Supplementary Tables S2 and S3) and defined 32 spatiotemporal intervals by partitioning eight developmental periods (P1–8) and four brain regions (R1–4), eliminating P3R4 (P3, late mid-fetal; R4, mediodorsal nucleus of the thalamus and cerebella cortex) due to insufficient data (Materials and Methods). We defined three different control datasets to reduce biases: (i) all brain-expressed proteins interacting with their physically interacting partners; (ii) common CNVs’ brain-expressed proteins interacting with their physically interacting partners, where the common CNVs were distinguished in the 1000 Genomes Project; (iii) all possible pairs between 22q11.21 CNV genes and human brain-expressed genes. We combined the physical PPI network with the human brain transcriptome to build up a brain-expressed human interactome, termed HIBE. After that, a static network was constructed by mapping CNV genes to the HIBE network. Next, the spatiotemporal expression data were integrated with the network, and the Spearman correlation coefficient (SCC) values were calculated. The interactions were certified only if the SCC was >0.5 (Materials and Methods). Finally, thirty-one networks were established.

### 3.2. 22q11.21 Co-Expressed Interacting Protein Pairs Are Enriched in the Early Mid-Fetal and Late Fetal Periods

**Figure 2:**
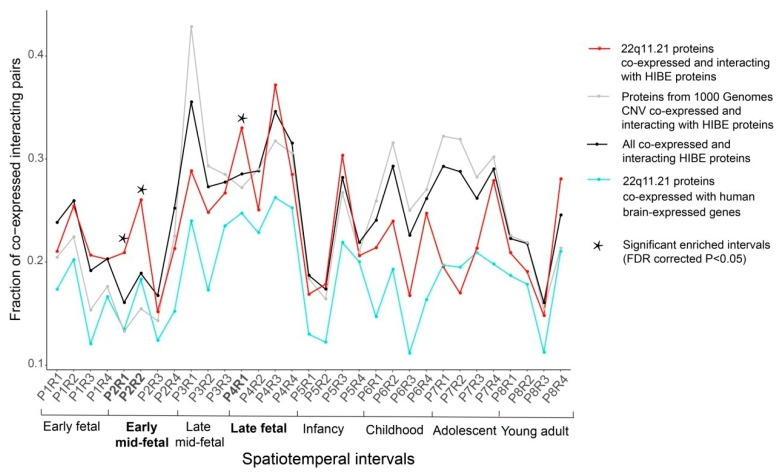
The 22q11.21 co-expressed interacting protein pairs are significantly enriched in three spatiotemporal intervals. The fractions of protein pairs from the 22q11.21 CNV co-expressed and interacting with HIBE proteins (red line), all co-expressed and interacting HIBE proteins (black line), proteins from the 1000 Genome Project CNVs co-expressed and interacting with HIBE proteins (dark gray line) and 22q11.21 CNV proteins co-expressed with all brain-expressed human genes (aquamarine line). Thirty-one spatiotemporal intervals of brain development are shown on the x-axis. 22q11.21 co-expressed interacting protein pairs are significantly enriched in spatiotemporal intervals (indicated by a star symbol) compared with the control networks. The statistical enrichments were calculated using Fisher’s exact test, and *p*-values were FDR-corrected for multiple comparisons.

To evaluate the statistically significant enrichment of connectivity for the 22q11.21 CNV, we calculated fractions of co-expression interacting pairs for 22q11.21 proteins and three control datasets (Materials and Methods). We identified that early mid-fetal and late fetal periods were significantly enriched in interacting pairs. After false discovery rate (FDR) correction for multiple testing, we identified significant enrichment in three intervals: P2R1 (P2: early mid-fetal; R1: parietal, temporal and occipital cortex; Fisher’s exact test, *p* = 0.00146), P2R2 (P2: early mid-fetal; R2: prefrontal and motor cortex; *p* = 6.6 × 10^−6^) and P4R1 (P4: late fetal; R1: parietal, temporal and occipital cortex; *p* = 0.018) (Figure 2).

### 3.3. Similarities and Differences between the Spatiotemporal 22q11.21 Networks

**Figure 3:**
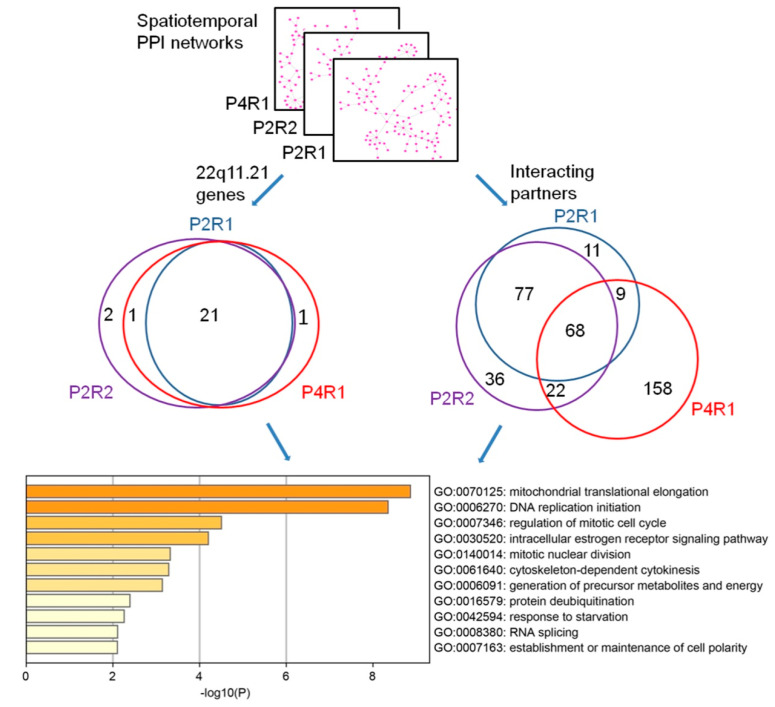
Functional convergence of the 22q11.21 spatiotemporal networks. The overlaps of 22q11.21 genes (left Venn diagram) and their co-expressed interacting partners (right Venn diagram) are across three significant spatiotemporal intervals. The top 11 significantly enriched biological processes’ GO terms of shared proteins are shown.

To assess the similarities among different spatiotemporal 22q11.21 co-expressed PPI networks, we measured their convergence by calculating the fraction of the shared proteins between these networks, P2R1, P2R2 and P4R1. We observed that 21 of the 26 (80.8%) 22q11.21 CNV proteins and 68 of their 406 (21.7%) co-expressed interacting partners were shared by all three networks (Figure 3, Supplementary Tables S4 and S5). Next, we performed functional enrichment on these shared CNV genes and shared interacting partners using Metascape (http://metascape.org) (Figure 3) and observed that the top three significant terms of the biological process were “mitochondrial translational elongation”, “DNA replication initiation” and “regulation of mitotic cell cycle”.

**Figure 4:**
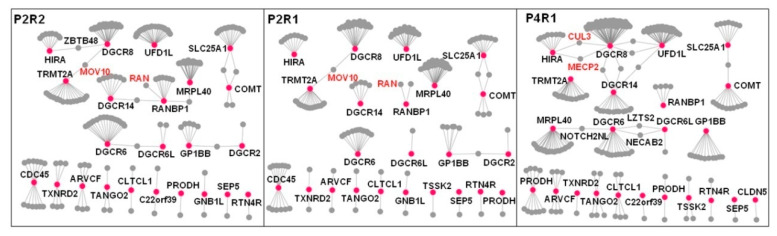
Difference between the 22q11.21 spatiotemporal networks. Spatiotemporal networks were compared across different brain regions within the same developmental period (P2R1 and P2R2) and across different development periods within the same brain region (P2R1 and P4R1). 22q11.21 genes are shown as red nodes, their co-expressed interacting partners as gray nodes and the PPIs between co-expressed genes at a particular developmental period as gray edges. The nodes that lost all edges were removed from the corresponding networks. Significant differences are observed across developmental periods and brain regions. The ANOVA statistics are shown in Table 1.

Next, we compared the connectivity of co-expressed interacting proteins either within the same developmental period (early mid-fetal P2) or within the same brain region (R1) to identify both topological and functional differences between spatiotemporal 22q11.21 networks. As noted, we identified three spatiotemporal networks with significantly enriched co-expressed PPI pairs across different brain regions (R1 and R2) within the same developmental period (early mid-fetal P2) and also across different developmental periods (early mid-fetal P2 and late fetal P4) within the same region (R1). Network changes were assessed by calculating the fractions of co-expressed interacting partners unique to one network and the fractions of co-expressed interacting partners shared by different networks (Figure 4, Table 1). We found statistically significant differences either between the same region within different developmental periods (P2R1 and P4R1, ANOVA test *p* = 2 × 10^−16^) (Table 1, Supplementary Table S6) or between different regions within the same developmental period (P2R1 and P2R2, ANOVA, *p* = 0.0186) (Table 1, Supplementary Table S7). These results demonstrate that the 22q11.21 network changes obviously across different developmental periods or brain regions.

### 3.4. 22q11.21 Networks Involved in the Regulation of Translation and DNA Replication

**Figure 5:**
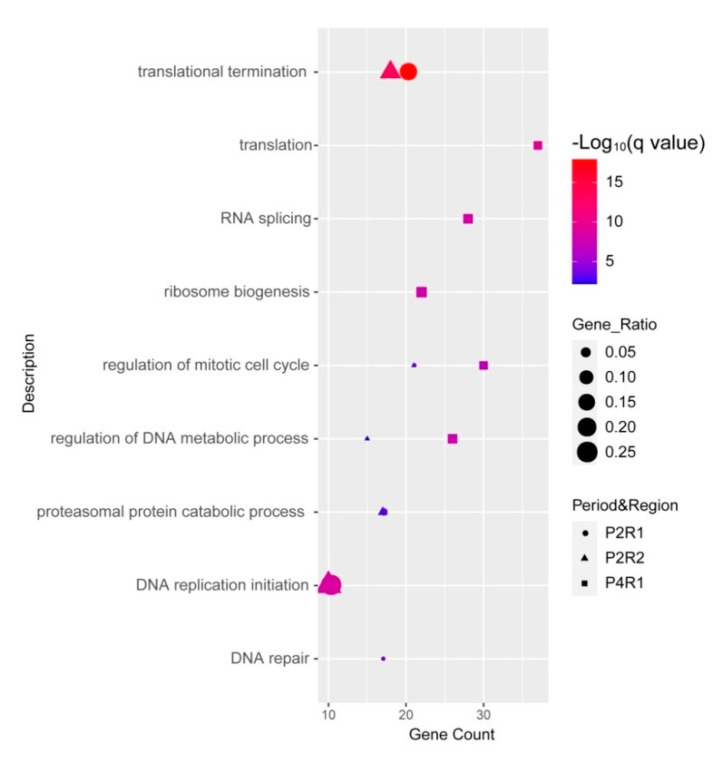
Functional analyses of proteins within three significant intervals, P2R1, P2R2 and P4R1. Dot plot shows significantly enriched GO terms of biological process for CNV proteins and their partners within three significant intervals.

Next, we investigated the biological functions of 22q11.21 proteins and their partners within three dynamic 22q11.21 networks, P2R1, P2R2 and P4R1. We used Gene Ontology (GO) and Kyoto Encyclopedia of Genes and Genomes (KEGG) to analyze the enrichment of the functional pathways (Materials and Methods). For 22q11.21 proteins and their partners from the P2R1 network, the top three significant terms of the biological process were “translational termination”, “DNA replication initiation” and “regulation of mitotic cell cycle” (Figure 5). Twenty genes were enriched in the term “translational termination”. There were ten genes enriched in “DNA replication initiation”, for instance, MECP2, CDK2, MCM3 and ORC1. Twenty-one genes, such as MECP2, BRCA2, CDK2 and RCC1, were enriched in “regulation of mitotic cell cycle” (Supplementary Table S8).

The top three significant terms for the biological processes involving 22q11.21 proteins and partners from the P2R2 network were “translational termination”, “DNA replication initiation” and “regulation of mitotic cell cycle” (Figure 5). Eighteen genes were enriched in the term “translational termination”, such as MRPL58, UPF1 and MRPL15. The term “DNA replication initiation” was enriched by ten genes, for instance, CDK2, MCM3 and CDC45. Twenty-one genes were enriched in “regulation of mitotic cell cycle”, such as RANBP1, PCNA and RCC1 (Supplementary Table S9).

For 22q11.21 proteins and their partners from the P4R1 network, the top three significant terms for the biological process were “translation”, “RNA splicing” and “ribosome biogenesis” (Figure 5). Thirty-seven genes were enriched in the term “translation”, such as DHX9, EGFR and ELAVL1. Twenty-eight genes, for instance, DDX5, DDX15 and ELAVL2, were enriched in the term “RNA splicing”. Twenty-two genes were enriched in the term “ribosome biogenesis”, such as DGCR8, DDX10 and DKC1 (Supplementary Table S10). 

We observed that 158 co-expressed and interacting partners of CNV proteins were only from the P4R1 network. These 158 co-expressed partners were not found in the P2R1 and P2R2 networks and were associated with “ribonucleoprotein complex biogenesis”, “RNA splicing via transesterification reactions” and “translation” (Supplementary Figure S1). Twenty-five genes were enriched in “ribonucleoprotein complex biogenesis”, such as DHX9, DDX10 and DKC1. Twenty genes, for instance, DDX5, DHX9 and ELAVL2, were enriched in “RNA splicing, via transesterification reaction”.

### 3.5. De novo Mutations Are Significantly Enriched in Spatiotemporal Networks

De novo mutations have recently been identified by exome sequencing and whole-genome sequencing from patients with psychiatric disorders [39,40] and have been observed to be potential genetic risk factors for psychiatric disorders [41,42]. Thus, we set out to investigate all 22q11.21 proteins and their interacting partners through the perspective of de novo mutations observed in psychiatric disorders (Materials and Methods). Previous studies collected de novo mutations from psychiatric disorders to generate disease- and phenotype-related gene sets [39]. Genes from the dynamic 22q11.21 networks were significantly enriched in ASD genes (FDR-corrected *p* = 1.0299x10^-6^). These genes were also significantly enriched in fragile X mental retardation protein (FMRP) target genes (FDR-corrected *p* = 1.0299 × 10^−6^) and voltage-gated calcium channel complex-related genes (FDR-corrected *p* = 1.42 × 10^−3^). There was no significant difference between the entire 22q11.21 network for developmental delay genes (FDR-corrected *p* = 0.224), long-term potentiation-associated genes (FDR-corrected *p* = 0.1024) and electrophysiology genes (FDR-corrected *p*-value = 0.254) (Supplementary Table S11).

### 3.6. Spatiotemporal Networks Identify Oivotal Co-Expression Partners in Developing Cortex

Within the P4R1 network, DGCR8 possesses the highest value of betweenness centrality among the CNV proteins, thus indicating that DGCR8 is a driver gene and adopts a central position within this network (Figure 4, Supplementary Table S12). Knockout of DGCR8 in zebrafish led to a decrease in brain size, and early developmental defects were observed as well [12]. Thus, we furthered our CNV investigation by focusing on DGCR8 and its interaction patterns across three spatiotemporal networks. Within the P4R1 network, two hub proteins, MECP2 and CUL3, interacted with DGCR8. As previously known, MECP2 interacts with DGCR8 to suppress Drosha-DGCR8-mediated miRNA processing, and it was shown to significantly reduce precursor and mature miRNAs [43]. Another P4R1 hub protein, CUL3, is also a DGCR8 partner and a core component of an E3 ubiquitin–protein ligase complex [44]. CUL3 mediates ubiquitination and degradation of target proteins [45]. Our observation suggested that CUL3 ubiquitin ligase promotes DGCR8 ubiquitination and proteasomal degradation.

DGCR8 interacts with MOV10 within P2R1 and P2R2 networks (Figure 4). As a component of the RNA-induced silencing complex (RISC), MOV10 is required for miRNA-mediated gene silencing [46,47]. In addition, DGCR8 interacted with ZBTB48 (Figure 4), which is a ZNF and BTB-containing protein [48]. Previous studies suggested that DGCR8 involves nucleotide excision repair (NER) to maintain genomic integrity during development [49,50]. ZBTB48 promotes rapid deletion of telomeric sequences to prevent telomeres from extreme elongation to protect genome integrity [48,51]. 

Within the P4R1 network, DGCR6 interacted with Leucine zipper putative tumor suppressor 2 (LZTS2) (Figure 4). LZTS2 negatively regulates microtubule severing at centrosomes and is necessary for centrosome spindle formation [52]. DGCR6 is involved in neural crest cell migration into the third and fourth pharyngeal pouches [53]. DGCR6 and DGCR6L share 97% identical amino acids [53]. Previous studies suggested that these two genes are candidate genes involved in the pathology of DiGeorge syndrome [54]. DGCR6 and MRPL40 (mitochondrial large ribosomal subunit protein 40) interacted with NOTCH2NL within the P4R1 network (Figure 4). While MRPL40 is involved in short-term synaptic plasticity [55], NOTCH2NL activates the Notch pathway by inhibiting interactions between Delta and Notch.

Within P2R1 and P2R2 networks, RANBP1 interacted with RAN (Figure 4). RAN is regulated by RANBP1 and plays an essential role in nucleocytoplasmic transport and mitosis [56]. A previous study showed that RANBP1 and RAN are involved in regulating axonogenesis [16]. Our results suggest that parietal–temporal–occipital lobes (R1) and the prefrontal and motor-sensory cortex (R2) are the primary regions for RANBP1 to modulate RAN during the early mid-fetal period.

### 3.7. Validation of the Interaction between DRCR8 and CUL3 by Immunoprecipitation and Liquid Chromatography-Tandem Mass Spectrometry (LC-MS/MS)

**Figure 6:**
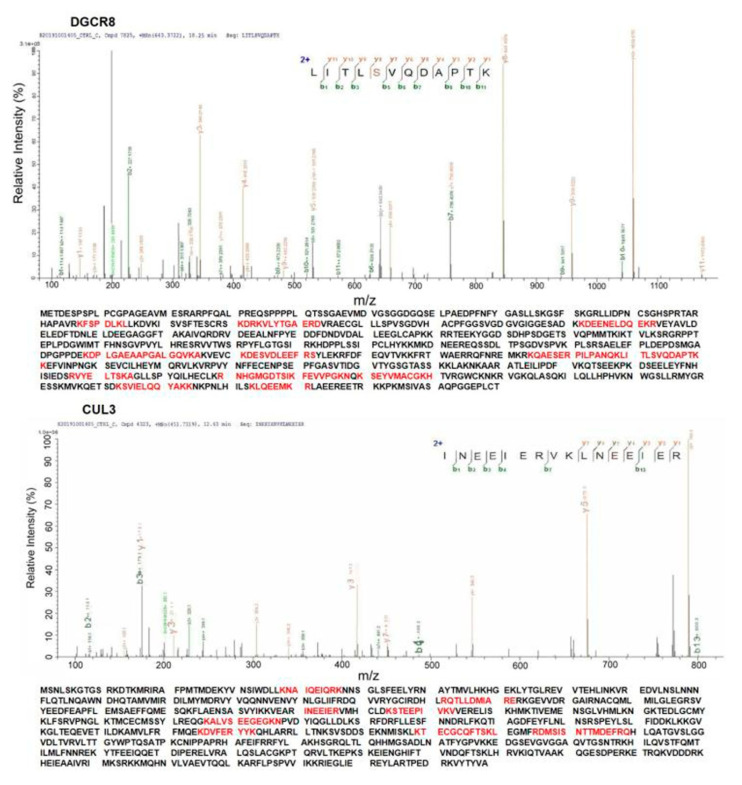
Investigation of interaction between DGCR8 and CUL3. DGCR8 and CUL3 were identified, and the amino acids marked with red color are peptides identified by immunoprecipitation (IP) and LC-MS/MS.

To confirm the interaction between DGCR8 and CUL3 in mammalian cells, we performed a co-immunoprecipitation (Co-IP) assay (Methods). The immunocomplexes from Co-IP were then detected by LC-MS/MS. More than five peptides were detected for DGCR8 and CUL3 (Figure 6). Each peptide was detected more than twice with high confidence. The interaction between DGCR8 and CUL3 was validated by LC-MS/MS (Figure 6).

## 4. Discussion

In the current study, we constructed a spatiotemporal network for the 22q11.21 CNV, a vital risk factor for psychiatric disorders, and carried out bioinformatics analysis for the CNV to identify the impacted brain regions, developmental stages and potential disease-related genes. Our spatiotemporal network analysis indicated that mid-fetal and late fetal periods were the critical periods for 22q11.21 CNV proteins to affect human brain development. Moreover, our study suggests that the frontal, parietal, temporal and occipital lobes are crucial regions affected by CNV genes. This result is also in accordance with previous reports that the frontal, parietal, temporal and occipital lobes were abnormal in 22q11.21 deletion carriers [57,58]. In sum, these results indicate that the 22q11.21 CNV plays a critical role in developing the human brain’s frontal, parietal, temporal and occipital lobes.

We identified that one CNV gene, *DGCR8*, is a driver gene in the parietal, temporal and occipital cortex (R1) during the late fetal period (P4). This result is consistent with the previous finding that homozygote DGCR8 mouse embryos demonstrated abnormal brain development [13]. One hub partner, MECP2, interacted with DGCR8. It was reported that MECP2 binds to methylated DNA, which activates or represses specific genes [59]. Previous studies reported that MECP2 is associated with severe neurodevelopmental disorders, including autism spectrum disorder and Rett syndrome [60,61]. DGCR8 is an essential component of the microRNA processing complex involved in the biogenesis of microRNA, and another work indicates that knockout DGCR8 could induce microcephaly [13]. Furthermore, previous works showed that MECP2 regulates the DGCR8/Drosha complex to suppress nuclear microRNA processing and dendritic growth [43]. Taken together, our results demonstrate that MECP2 interacted with DGCR8 in the parietal, temporal and occipital cortex to affect brain development during the late fetal period. 

DGCR8 interacts with another hub partner, Cullin 3 (CUL3). As a core component of the E3 ubiquitin ligase complex, CUL3 mediates proteasomal degradation. Previous studies proved CUL3 is a high-confidence risk factor for autism spectrum disorder and developmental delay [62,63]. CUL3 knockout mice showed autism-associated behavioral phenotypes. CUL3 is a critical component of E3 ubiquitin–protein ligase complexes involved in ubiquitination and degradation of target proteins [64,65]. The protein level of DGCR8 is decreased by ubiquitination [66,67]. Since the PPI interaction can be identified by Co-IP and liquid chromatography-tandem mass spectrometry (LC-MS/MS) [68], we then identified and validated the interaction between DGCR8 and CUL3 with high confidence by Co-IP and LC-MS/MS in our study. Our results suggest CUL3-mediated ubiquitination and degradation of DGCR8 to be involved in primary microRNA processing.

Furthermore, other essential CNV genes and partners were identified from dynamic networks. *MRPL40*, *DGCR6*, *DGCR6L* and *Ranbp1* are 22q11.21 CNV genes. In the parietal, temporal and occipital cortex (R1) during the late fetal period (P4), we observed that MRPL40 interacted with notch 2 N-terminal like A (NOTCH2NL), which is highly expressed in radial glia. NOTCH2NL promotes Notch signaling by interacting directly with NOTCH receptors. Previous works have demonstrated that NOTCH2NL is associated with the differentiation of neuronal progenitors [69,70]. MRPL40 has been shown to affect short-term synaptic plasticity through the regulation of mitochondrial calcium [55]. Our network analysis results indicate that MRPL40 might be involved in the NOTCH signaling pathway. Within the P4R1 network, DGCR6 and DGCR6L interacted with LZTS2. DGCR6 and DGCR6L associate with cell migration. LZTS2 regulates β-Catenin to be involved in microtubule severing, which is a significant mechanism for cell migration. Our results implicate that DGCR6 and DGCR6L may regulate cell migration via modulating LZTS2. Within P2R1 and P2R2 networks, Ranbp1 interacted with Ran. Ran is a Ran GTPase-binding and ras-related nuclear protein. Previous studies demonstrated that Ranbp1 influences the development of the cerebral cortex [16,71]. Ranbp1 interacts with Ran to influence Ran-guanosine triphosphate (GTP) gradients that triggered mitotic spindle assembly [72]. In addition, mice with a homozygous deletion of Ranbp1 also show microcephaly or exencephaly [15]. Our results suggest Ranbp1 affects human brain development in the parietal–temporal–occipital (R1) and prefrontal and motor-sensory cortexes (R2) during the early mid-fetal period.

After performing disease- and phenotype-related gene set enrichment analysis, we observed that genes from the spatiotemporal 22q11.21 network were significantly enriched in ASD genes, fragile X mental retardation protein (FMRP) target genes and voltage-gated calcium channel complex-related genes. Since Bernard J Crespi and Helen J Crofts showed that the 22q11.21 CNV is associated with ASD and schizophrenia [73], our results agree with previous works that discovered the 22q11.21 CNV as a significant risk factor for ASD [74,75].

## 5. Conclusions

In summary, we constructed dynamic 22q11.21 CNV networks to explore the pathological mechanisms of this CNV associating with psychiatric disorders. We identified that the frontal, parietal, temporal and occipital lobes are crucial regions for 22q11.21 genes to affect brain development during the early mid-fetal and late fetal periods. As a driver gene, *DGCR8* plays an important role in the parietal, temporal and occipital cortex during the late fetal period. Two vital hub partners, MECP2 and CUL3, interact with DGCR8. The physical interaction between DGCR8 and CUL3 was confirmed by liquid chromatography-tandem mass spectrometry (LC-MS/MS). Our results suggest that the DGCR8-dependent microRNA biogenesis pathway is crucial for the 22q11.21 CNV to be involved in psychiatric disorders. In addition, other CNV genes, such as *MRPL40*, *DGCR6*, *DGCR6L* and *Ranbp1*, may affect cortex development during the early mid-fetal or late fetal period. 

## Figures and Tables

**Table 1:** Summary of ANOVA test statistics.

ANOVA Tests	Sum of Squares	df	Mean Square	F	*p*-Value
P2R1 and P2R2	0.811	1	0.8114	5.971	0.0186 *
P2R1 and P4R1	7.302	1	7.302	257.8	2 × 10^−16^ ***

*** *p* < 0.001; * *p* < 0.05.

## Data Availability

The datasets used and/or analyzed during the current study are available from the corresponding author on reasonable request.

## Supplementary Materials

The following are available online at https://www.mdpi.com/article/10.3390/life11060514/s1, Figure S1: The significantly enriched biological process of 158 partners only from the P4R1 network, Figure S2: SDS-PAGE separation of the immunoprecipitated proteins, Table S1: Genes within the 22q11.21 copy number variation, Table S2: Developmental brain period from the BrainSpan related to Figure 1, Table S3: Four brain regions and the anatomical structures, Table S4: Shared 22q11.21 CNV genes for three spatiotemporal networks (P2R1, P2R2 and P4R1), Table S5: Shared co-expression interacting partners for three spatiotemporal networks (P2R1, P2R2 and P4R1), Table S6: Results of ANOVA test for interaction patterns of proteins from P2R1 and P4R1 networks, Table S7: Results of ANOVA test for interaction patterns of proteins from P2R1 and P2R2 networks, Table S8: Top 3 significant terms of biological process for proteins from P2R1 network, Table S9: Top 3 significant terms of biological process for proteins from P2R2 network, Table S10: Top 3 significant terms of biological process for proteins from P4R1 network, Table S11: Enrichment analysis of de novo mutation genes from 22q11.21 spatiotemporal networks, Table S12: Parameters of P4R1 network.

## Abbreviations

CNVs: copy number variants; PPI: protein–protein interaction; Co-IP: co-immunoprecipitation; LC-MS/MS: liquid chromatography-tandem mass spectrometry; 22q11.2DS: 22q11.2 deletion syndrome; FDR: false-discovery rate; LCRs: low copy repeats; RANBP1: Ran-binding protein 1; pri-miRNAs: primary miRNAs; ANOVA: analysis of variance; GO: Gene Ontology; KEGG: Kyoto Encyclopedia of Genes and Genomes; ASD: autism spectrum disorder; FMRP: fragile X mental retardation protein; IgG: immunoglobulin G; CBB: Coomassie brilliant blue; LC-MS/MS: liquid chromatography-tandem mass spectrometry.
