# Supplementary material for: Spatiotemporal 22q11.21 Protein Network Implicates DGCR8-Dependent MicroRNA Biogenesis as a Risk for Late Fetal Cortical Development in Psychiatric Diseases
Source: Life (Basel). 2021 May 31;11(6):514. doi: 10.3390/life11060514 (PMC8227527; doi:10.3390/life11060514)
Supplement: Supplementary file 1 [file life-11-00514-s001.zip › life-1240686-supplementary.pdf]

life

MDPI

Supplementary Materials

Spatiotemporal 22q11.21 Protein Network Implicates DGCR8-Dependent MicroRNA Biogenesis as a Risk for Late-Fetal Cortical Development in Psychiatric Diseases

1. Supplemental Figures

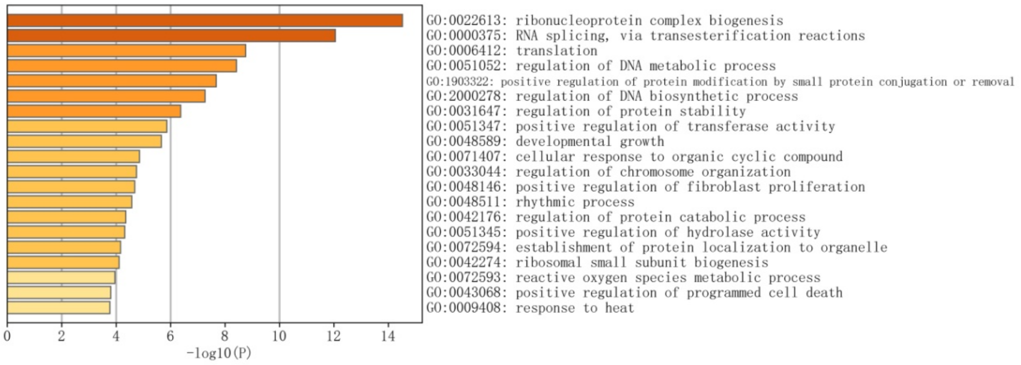

Figure S1. The significant enriched biological process of 158 partners only from the P4R1 network.

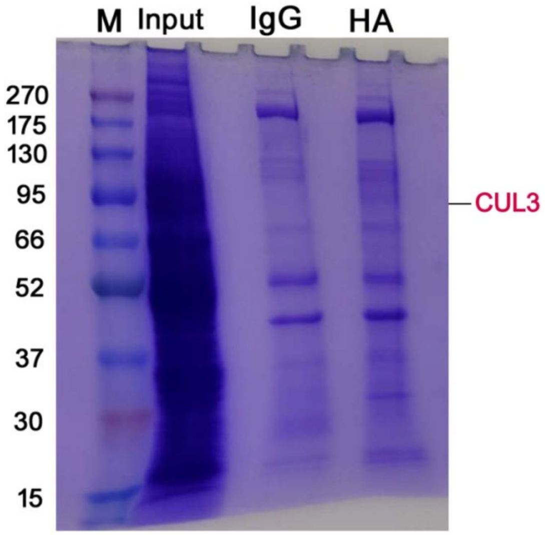

Figure S2. SDS-PAGE separation of the immunoprecipitated proteins. Cell lysate proteins were immunoprecipitated using control IgG or HA antibody. Several protein bands that are unique identified by LC-MS/MS were listed on the right side of the panel.

2. Supplemental Tables

Table S1. Genes within the22q11.21 copy number variation

Gene ID	Gene Symbole	Official Full Name	Position (hg38)	Size

8214	DGCR6	DiGeorge syndrome critical region gene 6	chr22:18,906,320-18,914,238	7,919
5625	PRODH	proline dehydrogenase 1	chr22:18,912,777-18,936,553	23,777
9993	DGCR2	DiGeorge syndrome critical region gene 2	chr22:19,036,282-19,122,454	86,173
8220	ESS2	ess-2 splicing factor homolog	chr22:19,130,279-19,144,651	14,373
23617	TSSK2	testis specific serine kinase 2	chr22:19,131,308-19,132,622	1,315
6576	SLC25A1	solute carrier family 25 member 1	chr22:19,175,581-19,178,736	3,156
8218	CLTCL1	clathrin heavy chain like 1	chr22:19,179,476-19,207,676	28,201
7290	HIRA	histone cell cycle regulator	chr22:19,330,698-19,431,696	100,999
64976	MRPL40	mitochondrial ribosomal protein L40	chr22:chr22:19,431,902-19,436,075	4,174
128977	C22orf39	chromosome 22 open reading frame 39	chr22:19,440,886-19,447,692	6,807
7353	UFD1L	ubiquitin recognition factor in ER associated degradation 1	chr22:19,449,911-19,479,193	29,283
8318	CDC45	cell division cycle 45	chr22:19,479,457-19,520,612	41,156
7122	CLDN5	claudin 5	chr22:19,523,024-19,527,545	4,522
5413	SEPTIN5	septin 5	chr22:19,714,503-19,723,319	8,817
2812	GP1BB	glycoprotein Ib platelet subunit beta	chr22:19,722,945-19,724,771	1,827
54584	GNB1L	G protein subunit beta 1 like	chr22:19,788,418-19,854,939	66,522
79680	C22orf29	retrotransposon Gag like 10	chr22:19,441,777-19,448,232	6,456
10587	TXNRD2	thioredoxin reductase 2	chr22:19,875,518-19,938,179	62,662
1312	COMT	catechol-O-methyltransferase	chr22:19,941,607-19,969,975	28,369
421	ARVCF	ARVCF delta catenin family member	chr22:19,969,896-20,016,823	46,928
128989	TANGO2	transport and golgi organization 2 homolog	chr22:20,017,014-20,065,358	48,345
54487	DGCR8	DGCR8 microprocessor complex subunit	chr22:20,080,232-20,111,875	31,644
27037	TRMT2A	tRNA methyltransferase 2 homolog A	chr22:20,111,875-20,117,392	5,518
5902	RANBP1	RAN binding protein 1	chr22:20,117,424-20,127,355	9,932
65078	RTN4R	reticulon 4 receptor	chr22:20,242,849-20,283,246	40,398
85359	DGCR6L	DiGeorge syndrome critical region gene 6 like	chr22:18,906,364-18,911,620	5,257

Table S2. Developmental brain period from the BrainSpan related to Figure 1

Stage	Description	Age	Developmental Period
1	Early fetal	8–9 PCW	P1
2	Early fetal	10–12 PCW	
3	Early mid-fetal	13–15 PCW	P2
4	Early mid-fetal	16–18 PCW	
5	Late mid-fetal	19–23 PCW	P3
6	Late fetal	24–37 PCW	P4
7	Early infancy	0–5 M	P5
8	Late infancy	6–11 M	
9	Early childhood	1–5 Yr	P6
10	late childhood	6–11 Yr	
11	Adolescence	12–19 Yr	P7
12	Young adulthood	20–39 Yr	P8

Table S3. Four brain regions and the anatomical structures

Brain Structure	Brain Region
Posteroinferior (ventral) parietal cortex (IPC)	R1
Primary auditory cortex (A1C)	R1
Posterior (caudal) superior temporal cortex (STC)	R1
Inferolateral temporal cortex (ITC)	R1

Primary visual cortex (V1C)	R1
The dorsolateral prefrontal cortex (DFC)	R2
Ventrolateral prefrontal cortex (VFC)	R2
Anterior (rostral) cingulate (medial prefrontal) cortex (MFC)	R2
Orbital frontal cortex (OFC)	R2
Primary motor cortex (M1C)	R2
Primary somatosensory cortex (S1C)	R2
Hippocampus	R3
Amygdaloid complex	R3
Striatum	R3
The mediodorsal nucleus of the thalamus (MD)	R4
Cerebellar cortex (CBC)	R4

Table S4. Sharing 22q11.21 CNV genes for three spatiotemporal networks (P2R1, P2R2, and P4R1).

No.	Gene ID	Gene Symbol
1	8318	CDC45
2	23617	TSSK2
3	5413	SEP5
4	7290	HIRA
5	7353	UFD1L
6	54487	DGCR8
7	2812	GP1BB
8	5902	RANBP1
9	64976	MRPL40
10	8220	DGCR14
11	8214	DGCR6
12	128989	TANGO2
13	8218	CLTCL1
14	6576	SLC25A1
15	1312	COMT
16	27037	TRMT2A
17	65078	RTN4R
18	5625	PRODH
19	85359	DGCR6L
20	10587	TXNRD2
21	421	ARVCF

Table S5. Sharing co-expression interacting partners for three spatiotemporal networks (P2R1, P2R2, and P4R1).

No.	Gene ID	Gene Symbol
1	54550	NECAB2
2	84062	DTNBP1
3	8480	RAE1
4	1762	DMWD
5	4173	MCM4
6	10474	TADA3
7	3396	ICT1
8	3665	IRF7
9	64928	MRPL14
10	79590	MRPL24
11	54148	MRPL39
12	51069	MRPL2

13	5713	PSMD7
14	6390	SDHB
15	79001	VKORC1
16	64210	MMS19
17	84445	LZTS2
18	4343	MOV10
19	3611	ILK
20	3985	LIMK2
21	6137	RPL13
22	155435	RBM33
23	26262	TSPAN17
24	55559	HAUS7
25	55630	SLC39A4
26	5111	PCNA
27	30968	STOML2
28	10105	PPIF
29	1072	CFL1
30	8928	FOXH1
31	3607	FOXK2
32	55611	OTUB1
33	284106	CISD3
34	57017	COQ9
35	388569	ZNF324B
36	10712	FAM189B
37	57104	PNPLA2
38	4998	ORC1
39	23594	ORC6
40	4172	MCM3
41	3609	ILF3
42	29855	UBN1
43	1104	RCC1
44	51258	MRPL51
45	122704	MRPL52
46	8449	DHX16
47	55388	MCM10
48	7185	TRAF1
49	55165	CEP55
50	10487	CAP1
51	1665	DHX15
52	51611	DPH5
53	3030	HADHA
54	55968	NSFL1C
55	11331	PHB2
56	51493	RTCB
57	10963	STIP1
58	6612	SUMO3
59	55622	TTC27
60	27339	PRPF19
61	22870	PPP6R1
62	64794	DDX31
63	115557	ARHGEF25
64	339230	CCDC137

65	23367	LARP1
66	55131	RBM28
67	9726	ZNF646
68	10498	CARM1

Table S6. Results of ANOVA test for interaction patterns of proteins from P2R1 and P4R1 networks.

**CNV**	**Genes**	**Interacting Partners in P2R1 and P4R1**				
**Entrez Gene ID**	**Official Symbol**	**Total Partners**	**Count of Unique to One Network**	**Freq of Network**	**Count of Shared by 2 Networks**	**Freq of Networks**
8214	DGCR6	24	19	0.7917	5	0.2083
5625	PRODH	2	1	0.5000	1	0.5000
9993	DGCR2	2	2	1.0000	0	0.0000
8220	ESS2	15	12	0.8000	3	0.2000
23617	TSSK2	5	4	0.8000	1	0.2000
6576	SLC25A1	10	10	1.0000	0	0.0000
8218	CLTCL1	7	7	1.0000	0	0.0000
7290	HIRA	13	12	0.9231	1	0.0769
64976	MRPL40	33	29	0.8788	4	0.1212
128977	C22orf39	1	1	1.0000	0	0.0000
7353	UFD1L	44	34	0.7727	10	0.2273
8318	CDC45	13	7	0.5385	6	0.4615
7122	CLDN5	1	1	1.0000	0	0.0000
5413	SEPT5	3	2	0.6667	1	0.3333
2812	GP1BB	9	9	1.0000	0	0.0000
54584	GNB1L	2	2	1.0000	0	0.0000
10587	TXNRD2	3	3	1.0000	0	0.0000
1312	COMT	12	12	1.0000	0	0.0000
421	ARVCF	3	3	1.0000	0	0.0000
128989	TANGO2	6	6	1.0000	0	0.0000
54487	DGCR8	83	77	0.9277	6	0.0723
27037	TRMT2A	22	21	0.9545	1	0.0455
5902	RANBP1	6	6	1.0000	0	0.0000
65078	RTN4R	2	1	0.5000	1	0.5000
85359	DGCR6L	2	2	1.0000	0	0.0000

Summary of ANOVA Test:

Groups Frequency of interactors	Count	Sum	Average	Variance
Unique to 1 network	25	22.0536	0.8821	0.0283
Shared by 2 network	25	2.9464	0.1179	0.0283

Result：

Source of variation	SS	df	MS	F	**p-value**
Between Groups	7.302	1	7.302	257.8	2×10^{-16}
Within Groups	1.360	48	0.028		
Total	8.662	49			

Table S7. Results of ANOVA test for interaction patterns of proteins from P2R1 and P2R2 networks.

**CNV**	**genes**	**Interacting partners in P2R1 and P2R2**

Entrez Gene ID	Official Symbol	Total Partners	Count of Unique to One Network	Freq of Network	Shared by Two Networks	Freq of Networks
8214	DGCR6	20	10	0.5000	10	0.5000
5625	PRODH	2	2	1.0000	0	0.0000
8220	ESS2	9	5	0.5556	4	0.4444
23617	TSSK2	2	1	0.5000	1	0.5000
6576	SLC25A1	4	4	1.0000	0	0.0000
8218	CLTCL1	1	0	0.0000	1	1.0000
7290	HIRA	6	3	0.5000	3	0.5000
64976	MRPL40	11	5	0.4545	6	0.5455
128977	C22orf39	2	2	1.0000	0	0.0000
7353	UFD1L	25	13	0.5200	12	0.4800
8318	CDC45	10	3	0.3000	7	0.7000
5413	SEPT5	2	0	0.0000	2	1.0000
2812	GP1BB	2	2	1.0000	0	0.0000
54584	GNB1L	2	2	1.0000	0	0.0000
10587	TXNRD2	1	1	1.0000	0	0.0000
1312	COMT	3	3	1.0000	0	0.0000
421	ARVCF	4	4	1.0000	0	0.0000
128989	TANGO 2	5	4	0.8000	1	0.2000
54487	DGCR8	28	8	0.2857	20	0.7143
27037	TRMT2A	8	2	0.2500	6	0.7500
5902	RANBP1	9	8	0.8889	1	0.1111
65078	RTN4R	1	0	0.0000	1	1.0000
85359	DGCR6L	2	2	1.0000	0	0.0000

Summary ANOVA Test:					
Groups		Count	Sum	Average	Variance
Frequency of interactors					
Unique to 1 network		23	14.555	0.6328	0.1559
Shared by 2 network		23	8.4453	0.3672	0.1359
Result:					
Source of variation	SS	df	MS	F	**p-value**
Between Groups	0.811	1	0.8114	5.971	0.0186
Within Groups	5.979	44	0.1359		
Total	6.79	45			

Table S8. Top 3 significant terms of biological process for proteins from P2R1 network.

Term	Description	Log(q-value)	Gene Count	Symbols
GO:0006415	translational termination	17.899	20	MRPL58, UPF1, MRPL46, MRPL15, MRPL2, MRPL37, MRPL51, MRPL39, MRPL50, MRPL16, AURKAIP1, MRPL47, MRPL14, MRPL40, MRPL11, MRPL9, MRPL1, MRPL24, MRPL52, MRPL21
GO:0006270	DNA replication initiation	−9.530	10	CDK2, MCM3, MCM4, MCM7, ORC1, ORC2, POLA1, CDC45, ORC6, MCM10
GO:0007346	regulation of mitotic cell cycle	−4.366	21	BRCA2, CDK2, RCC1, HSPA2, MECP2, ORC1, PCNA, PLRG1, PSMD2, PSMD7, RANBP1, CDC45, RAE1, CEP57,

				CTDSPL, CARM1, PHB2, CHMP2A, AURKAIP1, HAUS7, NSFL1C

Table S9. Top 3 significant terms of biological process for proteins from P2R2 network.

Term	Description	Log(q-value)	Gene Count	Symbols
GO:0006415	translational termination	–13.378	18	MRPL58, UPF1, MRPL15, MRPL2, MRPL37, MRPL51, MRPL39, MRPL50, MRPL47, MRPL14, MRPL40, MRPL32, MRPL11, MRPL9, MRPL1, MRPL24, MRPL52, MRPL21
GO:0006270	DNA replication initiation	–8.664	10	CDK2, MCM3, MCM4, MCM6, MCM7, ORC1, POLA1, CDC45, ORC6, MCM10
GO:0007346	regulation of mitotic cell cycle	5.88894	21	ASNS, BRCA2, CDK2, RCC1, ORC1, PCNA, PLRG1, PML, PSMD7, RANBP1, RDX, TUBA4A, CDC45, RAE1, CEP57, CTDSPL, CARM1, PHB2, CHMP2A, HAUS7, NSFL1C

Table S10. Top 3 significant terms of biological process for proteins from P4R1 network.

Term	Description	Log(q-value)	Gene Count	Symbols
GO:0006412	translation	–9.371	37	DHX9, EGFR, ELAVL1, GAPDH, GARS1, HNRNPD, HSPB1, MRPL58, ILF3, MOV10, NCL, RBM4, RGS2, RPL9, RPL13, RPS5, HNRNPR, MRPL28, LARP1, PPA2, RBMS3, MRPL2, MRPL4, MRPL51, DPH5, MRPL39, AURKAIP1, NAT10, ZC3H15, MRPL14, MRPL41, MRPL40, MRPL38, MRPL32, MRPL24, MRPL52, MRPL55
GO:0008380	RNA splicing	–8.382	28	DDX5, DHX9, DHX15, ELAVL2, ELAVL1, FUS, HNRNPD, HNRNPH1, HNRNPU, HSPA8, PPP2R1A, RBM4, SMN1, SRPK1, SRPK2, ESS2, DHX16, PRPF4, MPHOSPH10, HNRNPR, DDX17, PAPOLA, HNRNPUL1, PRPF19, RTCB, SRRT, RBM28, USB1
GO:0042254	ribosome biogenesis	–7.933	22	DDX10, DKC1, NOP2, NVL, RPS5, DDX18, BTRC, MPHOSPH10, NSA2, DDX17, DHX30, GTPBP4, DROSHA, GNL2, PAK1IP1, NAT10, BRIX1, DDX24, TENT4B, DDX31, NOL10, UTP23

Table S11. Enrichment analysis of *de novo* mutation genes from 22q11.21 spatiotemporal networks.

Number of Genes within 22q11.21 Spatiotemporal Networks	Number of Autism *de novo* Mutation Genes	Number of Autism *de novo* Mutation Genes in Networks	Fisher Exact p-Value	Benjamini-Hochberg adj p-Value
994	239	34	2.814×10^{-7}	1.0299×10^{-6}

Number of Genes within 22q11.21 Spatiotemporal Networks	Number of FMRP *de novo* Mutation Genes	Number of FMRP *de novo* Mutation Genes in Spatiotemporal Networks	Fisher Exact p-Value	Benjamini-Hochberg adj p-Value
994	839	80	3.433×10^{-7}	1.0299×10^{-6}

Number of Genes within 22q11.21 Spatiotemporal Networks	Number of Voltage-Gated Calcium Channel Complexes *de novo* Mutation Genes	Number of Voltage-gated Calcium Channel Complexes *de novo* Mutation Genes in Spatiotemporal Networks	Fisher Exact p-Value	Benjamini-Hochberg adj p-Value
994	206	23	0.0007124	1.42×10^{-3}

Number of Genes within 22q11.21 Spatiotemporal Networks	Number of Developmental Delay *de novo* Mutation Ggenes	Number of Developmental Delay *de novo* Mutation Genes in Spatiotemporal Networks	Fisher Exact p-Value	Benjamini-Hochberg adj p-Value
994	1291	74	0.1867	0.224

Number of Genes Within 22q11.21 Spatiotemporal Networks	Number of Electrophysiology Genes	Number of Electrophysiology Genes in Spatiotemporal Networks	Fisher Exact p-Value	Benjamini-Hochberg adj p-Value
994	213	6	0.254	0.254

Number of Genes within 22q11.21 Spatiotemporal Networks	Number of Long-Term Potentiation-Associated Genes	Number of Long-term Potentiation-Associated Genes in Spatiotemporal Networks	Fisher Exact p-Value	Benjamini-Hochberg adj p-value
994	157	13	0.06824	0.10236

Table S12. Parameters of P4R1 network.

Entrez Gene ID	Name	Betweenness Centrality	Eccentricity
8214	DGCR6	0.63989899	3
6576	SLC25A1	0.61403509	3
7290	HIRA	0.1207001	5
64976	MRPL40	0.80808081	5
7353	UFD1L	0.49880895	5
1312	COMT	0.78947368	3
54487	DGCR8	0.81570979	3
85359	DGCR6L	0.04494949	5

Note: PRODH, TSSK2, CLTCL1, C22orf39, CLDN5, SEPT5, GP1BB, TXNRD2, ARVCF, TANGO2, TRMT2A, RANBP1, RTN4R were excluded because of isolating from the network.
